# Sequence Analysis of the Mouse RAG Locus lntergenic Region

**DOI:** 10.1155/1998/54045

**Published:** 1998

**Authors:** F. E. Bertrand III, S. L. Olsona, D. A. Martin, G. E. Wu

**Affiliations:** 1 Wellesley Hospital Research Institute and the Department of Immunology University of Toronto Ontario Canada; 2 Wellesley Hospital Research Institute 160 Wellesley St. E. Toronto ON MHY 1J3 Canada

**Keywords:** RAG-1, RAG-2, lymphopoiesis, regulation, promoter, enhancer

## Abstract

The recombination activating genes RAG-1 and RAG-2 are highly conserved throughout evolution and are necessary and essential for the DNA rearrangement of antigen-receptor gene segments. These convergently transcribed genes are expressed primarily by developing B and T lineage cells. In addition, recent data suggest that the RAG locus can be reactivated in mouse germinal center B cells. Despite these well-defined patterns of expression, little is known about mechanism(s) regulating transcription of the RAG locus. Experiments with a mouse fibroblast line stably transfected with a genomic fragment of the RAG locus suggest that the intergenic region between RAG-1 and RAG-2 may contain information modulating RAG transcription. In order to begin testing this hypothesis, we have sequenced the 7.0-kb RAG intergenic region of the mouse. The sequence did not contain open reading frames larger than 60 amino acids. Analysis with GCG software identified several potential transcription-factor binding sequences within this region. Many of these are associated with transcriptional regulation of the Ig locus.


*Developmental Immunology*, 1998, Vol. 5, pp. 215–222
Reprints available directly from the publisher
Photocopying permitted by license only

© 1998 OPA (Overseas Publishers Association)
Amsterdam B.V. Published under license
under the Harwood Academic Publishers imprint,
part of the Gordon and Breach Publishing Group
Printed in Malaysia

### Sequence Analysis of the Mouse RAG Locus Intergenic Region

F. E. BERTRAND III*[†], S. L. OLSON[a], D. A. MARTIN[a] and G. E. WU[a]

*Wellesley Hospital Research Institute and the Department of Immunology, University of Toronto, Ontario, Canada.*

*(Received 7 April 1997; In final form 28 April 1997)*

The recombination activating genes RAG-1 and RAG-2 are highly conserved throughout evolution and are necessary and essential for the DNA rearrangement of antigen-receptor gene segments. These convergently transcribed genes are expressed primarily by developing B and T lineage cells. In addition, recent data suggest that the RAG locus can be reactivated in mouse germinal center B cells. Despite these well-defined patterns of expression, little is known about mechanism(s) regulating transcription of the RAG locus. Experiments with a mouse fibroblast line stably transfected with a genomic fragment of the RAG locus suggest that the intergenic region between RAG-1 and RAG-2 may contain information modulating RAG transcription. In order to begin testing this hypothesis, we have sequenced the 7.0-kb RAG intergenic region of the mouse. The sequence did not contain open reading frames larger than 60 amino acids. Analysis with GCG software identified several potential transcription-factor binding sequences within this region. Many of these are associated with transcriptional regulation of the Ig locus.

*Keywords:* RAG-1, RAG-2, lymphopoiesis, regulation, promoter, enhancer

### INTRODUCTION

Genes encoding antigen receptors are assembled through somatic DNA rearrangement of the gene segments encoding the variable portions of the immunoglobulin molecule (see Lewis, 1994, for review). Typically, this process occurs during discrete stages of lymphocyte development in either the bone marrow for B cells or the thymus for T cells. The recombination activating genes, RAG-1 and RAG-2, are necessary and essential for this process (Oettenger et al., 1990; Mombaerts et al., 1992; and Shinkai et al., 1992).

The coordinately transcribed RAG-1 and RAG-2 genes are usually expressed together. Only in the chicken bursa (RAG-2 only) and in the mouse brain (RAG-1) are one of the RAG genes expressed without the other (Chun et al., 1991; Takeda et al., 1992). The expression of the RAG genes varies throughout lymphopoiesis. In murine B lineage development,

*Corresponding author.
†Present address: Wellesley Hospital Research Institute, 160 Wellesley St. E., Toronto, ON MHY 1J3, Canada.

high levels of RAG are found in the earliest stages of B lineage development when heavy-chain rearrangement initiates ($B220^+$ $CD43^+$ cells) and then decreases through the cytoplasmic $\mu^+$ stage. RAG levels rise again during the onset of light-chain rearrangement (Li et al., 1993). A similar pattern is seen in human B-lineage development (Ghia et al., 1996). In more mature B cells, the RAG locus is inactive. However, RAG expression has recently been demonstrated in germinal-center B cells of mice that have undergone immunization (Han et al., 1996; Hikida et al., 1996), suggesting a role for the RAG proteins in later aspects of B-lineage development, such as receptor editing.

Several studies have indicated that RAG transcription can be modulated through pathways involving protein kinase A (PKA), protein kinase C (PKC), and cAMP. Increases in cAMP result in an increase of RAG transcription, acting through the PKA pathway. Induction of the PKC pathway will decrease RAG transcription (Menetski and Gellert, 1990; Casillas et al., 1995). However, the *cis*-acting elements involved in this modulation of RAG locus are unknown.

Using the fibroblast line L4, which contains a genomic fragment of the RAG locus under the control of the SV2 promoter (Schatz et al., 1989), Dobbeling and colleagues showed that transcription from this fragment can be influenced via the PKA and PKC pathway in the same way that these second messengers influenced transcription from the endogenous RAG locus in a pre-B cell line (Dobbeling et al., 1996). Moreover, the genomic fragment in the L4 fibroblast line was lacking the region 5′ of RAG-2 (Schatz et al., 1989). This result suggests that elements modulating RAG locus transcription through these second messenger pathways lie 5′ of RAG-1 and/or in the intergenic region between the two genes.

On the basis of these data, and because the RAG genes are relatively close together (7.0 kb in the mouse, 2.6 kb in the zebrafish), we hypothesized that the intergenic region in between RAG-1 and RAG-2 may contain sequence element(s) involved in coordinately modulating transcription of the RAG locus. In order to investigate this possibility, we have sequenced the murine 7.0-kb RAG intergenic region. Analysis with GCG software has revealed a variety of potential regulatory elements in this region.

### MATERIALS AND METHODS

### Cloning of the Murine RAG Intergenic Region

The clone pJH493, which encompasses most of RAG-1, the intergenic region, and RAG-2, was the kind gift of Dr. J. Hesse, NIH. Two overlapping fragments of the intergenic region were subcloned from the pJH493 plasmid. Fragment 1 (4.0 kb) was generated by digesting pJH493 with Bgl II and cloning the fragment into the Bam H I site of pBluescript (Stratagene, La Jolla, CA). Fragment 2 (3.5 kb) was generated by digesting the pJH493 with EcoR V and subcloning into the Sma I site of pBluescript (Stratagene, La Jolla, CA).

### Sequence Analysis

Single-stranded sequence of the Fragment 1 and Fragment 2 clones was obtained using a Pharmacia A.L.F. automatic sequencer (HSC Biotech. Centre, U. Toronto). Sequences were assembled using DNAStrider and analyzed for transcription binding-factor motifs using the TFSITES database and GCG software. Homology searches were done using the NIH Blast algorithm for searching GenBank. This sequence has been deposited in GenBank under the accession number U96151. GRAIL software developed by ORNL (Oak Ridge National Laboratory, Oak Ridge, TN) was used to assess the region for potential open reading frames.

### RESULTS

### Sequence of the Murine RAG Intergenic Region

The murine RAG intergenic region ($\sim$ 7 kb) was subcloned from the pJH493 clone of the RAG locus, as two overlapping fragments 4.0 and 3.5 kb, respectively (Figure 1). Single-stranded sequence of each fragment was obtained as described in Materials and Methods. This sequencing revealed the RAG

intergenic region (defined here as beginning immediately 3′ of the published 3′ UTR sequence of RAG-1 and RAG-2) to be 7004 base pairs in length. The intergenic region is illustrated in Figure 1 and has been deposited into GenBank under the accession number U96151. Figure 2 lists the sequence. Three-phase amino acid translation analysis of this region failed to reveal any open reading frames. The GRAIL computer algorithm, designed to detect open reading frames (ORFs) in genomic sequences was also used to examine this region for the ability to encode a gene. In agreement with the translation, no potential ORFs were identified. This result is consistent with previous Northern analysis of this region (Schatz et al., 1989).

### Potential Transcription-Factor Binding Sites of the Murine RAG Intergenic Region

GCG software and the TFSITES database were used to examine this region for potential transcription-factor binding sites. By using a mismatch of zero, the consensus sequence for a variety of transcription-factor binding sites were identified. Many of these are

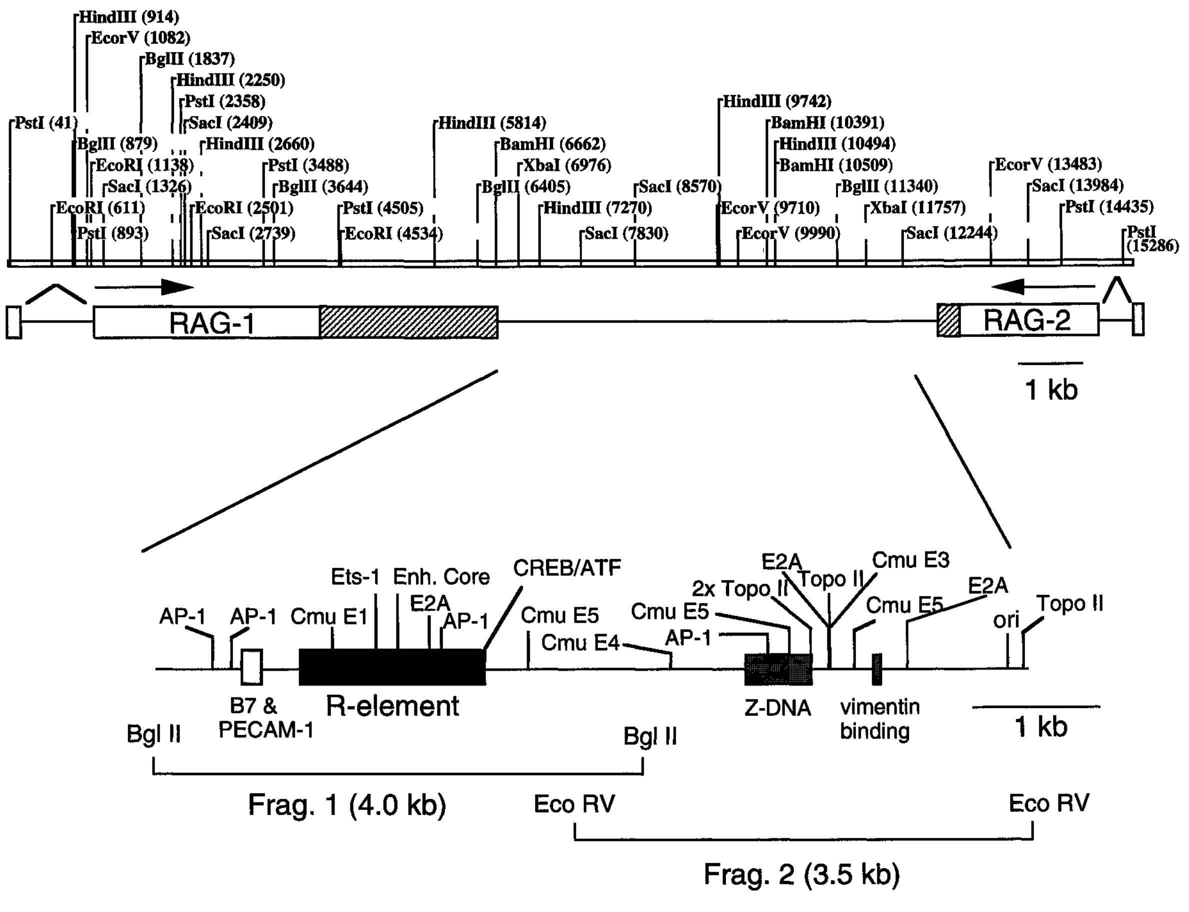

FIGURE 1 The murine RAG locus, illustrating the intergenic region. For RAG-1 and RAG-2, the open box reflects coding regions and the hatched boxes are the 3′ UTRs. The expanded section is the sequenced intergenic region (accession number U96151). Potential transcription binding sites are shown above the line, and shaded boxes indicate repetitive genomic features. The top portion is a restriction map of the mouse RAG locus compiled from accession numbers M29475 (RAG-1), M54796 (RAG-2), and U96151 (the intergenic region). The numbers begin with M29475 and are numbered consecutively through RAG-2. Not included in the restriction map are the two introns immediately following 3′ of the 5′ UTRs of RAG-1 and RAG-2, indicated by inverted V's. Also shown are the locations of subclones Fragment 1 and Fragment 2.

known to be involved in the regulation of genes expressed in lymphopoiesis. Of note, are the AP-1 sites and the single CREB/ATF site (Figure 1), because these transcription factors have been shown to be involved in pathways regulating RAG transcription (Dobbeling et al., 1996). The locations of these sites are plotted in Figure 1 and listed in Table I. Binding sites for Cmu E5 and topoisomerase II are included in the list because of their known role in regulating the IgH enhancer, although the sequences for these deviate from their respective consensus by one or two base pairs (reviewed in Ernst and Smale, 1995).

### Repetitive Elements Found within the RAG Intergenic Region

A search of GenBank using the NIH BLAST algorithm revealed that the murine RAG intergenic region contains regions of homology with previously reported repetitive elements in the mouse genome. The location of these elements is shown in Figure 1. Base pairs 624-824 have sequence identity with B1-type repetitive elements found in the B7 and PECAM-1 promoters (Selvakumar et al., 1992; Almendro et al., 1996). From bases 1124-2524 is a region that is similar to murine R-elements. There are nearly 100,000 copies of R-elements in the murine genome,

1 tatttggaat aaaagtttaa gatctgaaaa taacaggcgt tgtgatttat attggtgcta tatcatctca tatcaattat gttgactgcc catacttaa cattatctcc cttatttgaag
121 gacataaaaa tgaacttggt gactgtgata cttagtctca gttgtcaact ggagaaatag catcatccga gagtcaggac tctggcgtag actgtggga gcatcctttc attgggctaac
241 caatgggtct ctgtcagtgg cattgttcct cgtgtgggat ccttgcctgg gtgaaccggg agaaagtgag ctacccaagc attcatggct ctctgcttt cctgacctgt tacttcgagtt
351 catgggcccc tgtcttgctg tgatggatgc ttttgaattg tgaggtgaaa taaatctgtt tgcact**tgag** **tcac**tgggga cagtttatca cagcattat ggaaagtttg aaccaccttttg
481 tttcaagggt actagattag ttaagaatat aatttatcat aatgtagggt gaaagatttt ggttccagat aatgtaaaca gtaaaacctt aaacatgct tttaagaatc ttctagagatt
601 cttataancc aaaacctg**tg** **attaa**aatta tgattttttt tttcagttgc ccttggccac ttacattatt ggcaaaacta gtcagatccc aggctcatt gcccactgct agcaataataa
721 tgaaacaggc atggtggaaa acacttgtag tctcaactct tagggaggct gatgcagaag ,aattgtgaat ccaaggctga cttactgggc acaaaagac aaaatcaagc caactaaatag
841 atacagacac aaaaccaaat ccaaaccaga tagttatcat ttaccaagct tctttttttc cgtggtgtca agtaaagtgg tgtaaaacaa ctttacata tgttgccaca tcctatccttt
961 tgaactaggt gcttctgtga tttccatttc acatgtggtc agggaagtat atcttagcca gaatcatatc tttaccatct actccgtcta gctcatctt tgttgagact cttccctttac
1081 ttaactcgac acacttcacg atccctttca ctttggactg aaatttagtc tctgtctata tatagctgta ccagttctta aaatactgta cttatagtc caatagctta tccccaagacc
1201 taattgcctg tattgtttta atttttttccc attattttct tcatttacta ttcaaattca aatgctatcc tgaaagtccc ctacactctc cccctgccc tgctccccaa cccacccactc
1321 ccacttcatg gccctggcat tcccctgtac tggggcatat aatctactca agaccaaggg tctctcctcc caat**gatggc** **cg**attaggca tctttcttg ctacatatgc agctagagaca
1441 tgagctctgg gggnactggg tagttcatat tgttgttcct cctatacgat tgcagacccc ttcagctctt tgggtacttt ctctggctcc tccattggg ggccctgtgc tccgtccaata
1561 catgaccgtg agcacccact tctgtatttg ccaggcactg gaatagcctc acaagagaca gctatatcag ggtcctgtca gcaaaatctt gctgtaatt gcccttatat taatttattat
1681 ctcctttcag catcatttta gaacatagta acaagttaac ccctagtgat **acttccgg**ac ctgacctttt gagtatttga atattaatcc tgcttttga aaatgtagta tatccttactt
1801 aaaagtaagg ccca**gtggaa** **tg**ttttcct ttatgctatc atttctaact actccagcct ctattttatt ttatttgaag attcatacat gagtacact gtatttgcat aattttttgttt
1921 tgctttgttt gttttttttat tagatatttt ctttatttac atttcaaatg ttaccacctt tccttgttta ttctctgaaa acccccttatc tcacccctc cccctgctca tcctgttcccc
2041 taccctggca ttcccctaca ctgggccatc cagccttcac aaggttaagg gcttcncctc ccactgatgt ccaacaggcc atcctctgct acatat**gca** **gctg**gagcca tgtgtccctct
2161 ttggttggta gt**ttagtcac** tgggagctct ggggtattgg ttggttcata ttgttgtccc tcctgtgggg ctgtaaacct cttcagcttc ttcagtcag ttctctagct cctccattggt
2281 ggcctcgtgc tcagtccaat ggttggctga aagaatccac ctcggtattt gtcaggctct ggtagagcct ctcaggagat ggctatatca ggctcctgt cagcaagcac ttgttggcttt
2401 taaaccctac tctctccttt aacttttcct ttatcttctt ccaactgccc ctctcaaatg tataatttct tgtatattta tttttgtttt ttgatgtct tccagtttat tcagtttactt
2521 tgagtaggct taatagatgt taaaccatgg gcaataacat tagaagaaag ttcacatggt taggagagca cagagaaaac acaaaatgca gcct**tgacg** **t**gagagatga gtacccagagg
2641 aaacagattt ctttacccat ctttttttctt taatctctct cataattgct tcttgactgt attttatttt acacaaacac acacacacac acacacaca cacacacaca tcatataccac
2761 tatcctatac acaaactact aagtaaagct agtgtttccc ttatt**tgcat** **gtgt**ttaggg atgattctat aggattggtg tctcatctct gaagatacc accaccacca ccaccaccacc
2881 accaccacca ccaccaccac caccggcagc ggcagcagca gcagcacaac caccaccacc agcaccacca tcaacacctt aatacaaaac cacatagaa gtggttctcc tttcctcagca
3001 gccattgact gcctgtatct cagaggaaat ggggaccttg gaatttcctc cacccatgtt agtatggtca ttcatgtggg ctttgcagga cttctttgg gtagccatat tattgagagtt
3121 catgggagca acatccctgt cctgtccaga ggaaactacc atgcagcaga tgttctggtc cacaagctct tataatctct ccatctcttt ttttccaga gattttccct gagatttagtt
3241 tttagggttg tgctatagat gccccatttg agaggctggg tgcaccatgg ttactaattt tctgcattta ttttgactag ttgaggatat cagcaatag tttctgtctg ctgcaaaaaga
3361 agctttttttc atgtggtgtg agagttgcat tgatttatgg gtagaaggac ggacatttag aaggcaatcg aaaattactt cagtaatatg caataataa atccacctct aagctcagtga
3481 catctctaaa catgggcttt tggccaggtt tacaatatca ggcatgagtt tccatcaatt gagtgggctt tagttccaat tagacaactg ttagtttcc cctgagtttc attcttgcaca
3601 gctaggatat cttgctagtg ccatcatttt tttggttctg aaactttaca gctatataag actgttgact aaatttctcc tttggcagat catcagact ctattgtaag ttttatgggct
3721 gcttcttcta agcaggcgat gtatacttgg tactgagata cagtggcaag tagcatagtt ttggttcaca aggagctagg catcaagtta tcaacagta agactaatga gggctcatgct
3841 ctactgtgga ggagaaaaaa cattagatgc aattataata gataagtata aaaaagtaca gtgtgacagt aggatttaaa actggggagt ctgaaaaaa aaa**accacct** **g**ggaagaaaga
3961 atgagtgggt tgaagttagg tcagttacct agagaagaca gcctctgggg atccgaggca ggaaagcatt cagagggttt gggaatttaa gaaagacaa gttgacagta acagactggac
4081 gttggggctg gactgctgga gaatcaggca gaagctttgt gctgtcggat ccagtggaca cccccttgggc ataaaaggaa accatgttgg agtttgagg atatgatgca gtctaaaccac
4201 aatggaacaa ggcacaagag aatgctctag gaaagtatta atctcaagga ctctggccag gtttgagaaa ggttagggaa ctggagacaa gtttgagca tgccctttta cttcagtggta
4321 caccgtctac aagttaatgt ctcattctct cagacaacag ttcccaagcc caggtgggaa gagatggagg aggattattc atgcgtaaaa gtcccacct ttttatgaag ttgtttattta
4441 cccgttcttt ccccttttggt cttatagaaa tttccttttct ccttttattg ctttttattc tttcctgaag atgacaatat atttctgatg taaaaacaa aacaaaacaa aacaaaaacaa
4561 aaaaaaaaaa ctattcattc tgggctgggg agatagctga tttgttatag tgctttgcat gccaggagcc cgagtttgat cttcagaacc cacctaaaa aaccaaggtg ttagaacattc
4681 tcttaatgtc agtgctggga gggagagaca aggccatttc tgatgtccag ctagtctaat ctac**ttagtc** **ag**ttccagga cagtgaggga ctgtcataa aaatggtgga ggtcatctgaa
4801 gaatgacacc catgggtgtc tttgtgactt ccagaggcac aagcacacat tcacacactc tcata**tacag** **gtgt**acacac tcatg**caaat** **ggacataca** **c**agaggaa**ca** **cgtgtatgtac**
4921 **ac**agacggac agacacaccc taatttactg aatgccaaga tctgtgtatt tctacactga agtctgatca aagtctagtg aactggtcca tctctacaa ctattttcca tctttgcccct
5041 aacaagttca tttttttttt gttgtcttaa atgatatggt catcctttgc aacacagatt tccccttttaa agtgattact atacattttg taattactt caaatggctt agtctaaaggt
5161 tttgttatct ttgccacaat aattcttcag cagaggtgat ataaaatagt ctgaggtact ttggccccat tctgaagttt tgtcagtata ttccaggtt agaaaagagt ttgtatttgca
5281 tgggcagcct tttctggggc tcaggtacat **acagatg**tgc tttgtatctt ttcttccctg tccttggcct ctttctaagc gagacaacaa attctctag agatgatttt ttttttttgca
5401 ggtcccatga tatttaatgt ggctcttgga attaaaaaag tgtgtgtatg tgtgtgtgtg tgt**gtgtgta** **tccatatg**tg tgcgt**ggtca** **tgtggc**tgt gga**tgcatgt** **gt**acatatggg
5521 catgtggatg tggatatagg ccagagacca gcttcaaatg ttgtttctct **tcagctgc**ca tccaccttgt tttatttttt gataaagaat gttgaacaa cgatggggac tcaggtacaga
5641 ctgcgtttag ggaaggaggt ggtggaacta aagaatcatg tcaagccaca atgagaaatt tggtgctttt cctacgagaa actaagactt ttatggaat atgaaagttt tcttttttattg
5761 attacatatt ttttgttata atttctgtat tcagtggaag cagttcagga cttagcagta gagcctcctt gagagtgata gtcccaaacc tttgtctga gctcatgttc atatttcactt
5881 ttcacttaaa gtggtaatgc catacttcag agctgcacag tattggacct tgaatgacat gtgactattt ccagagatga caactgtctt taaaagatg agggactatg gctaagcattt

FIGURE 2 Sequence of the murine RAG locus intergenic region (accession number U96151). Boldface sequences are the potential transcription-factor binding sites listed in Table 1.

TABLE I Transcription-Factor Binding Motifs in the Murine RAG Intergenic Region†

| Transcription Factor | 5′ end | Sequence |
|---|---|---|
| AP-1 | 427 | TGAGTCAC |
| AP-1 | 619 | TGATTAA |
| Cu E1.2 | 1395 | GATGGCCG |
| Ets-1 | 1731 | ACTTCCGG |
| Enhancer core | 1815 | GTGGAATG |
| E2A | 2137 | GCAGCTG |
| AP-1 | 2173 | TTAGTCAC |
| CREB/ATF | 2615 | TGACGT |
| Cmu E5 | 2806 | TGCAtGTGT |
| Cmu E4 | 3943 | ACCACCTG |
| AP-1 | 4745 | TTAGTCAG |
| Cmu E5 | 4866 | TaCAGGTGT |
| Topoisomerase II/Rev | 4886 | CAAATGgAcATACAC |
| Topoisomerase II/Rev | 4908 | CACgTGtATGTACAC |
| E2A | 5311 | ACAGATG |
| Topoisomerase II | 5464 | GTGTgTATcCATATG |
| Cmu E3 | 5489 | GTCATGTGGC |
| Cmu E5 | 5503 | TGCAtGTGT |
| E2A | 5572 | CAGCTGC |
| Topoisomerase II | 6888 | CTCATAAgTATAAAC |

†Indicated are selected transcription-factor binding sites and the location of the 5′ end of each sequence. These sites correspond to the sequences in boldface in Figure 1. Numbering refers to GenBank accession number U96151. Mismatches from the consensus are indicated by lowercase letters.

found in association with a variety of genes, including the $\beta$-globin locus and the Ig light-chain loci (Gebhard et al., 1972; Gebhard and Zachau, 1983). It is interesting to note that many of these repetitive elements are associated with the promoters of a variety of genes, and that the potential transcription-factor binding sites seem to cluster around the location of the repetitive elements.

### Regions of Possible Matrix Attachment within the RAG Intergenic Region

The intergenic region from bp 4500 to 6800 contains sequences associated with matrix attachment and unusual DNA structures. This region contains a sequence with ~60% homology with the consensus sequence for the binding site of the intermediate filament vimentin (Figure 1). This protein is thought to selectively anchor areas of GC-rich DNA to areas of matrix attachment (Wang et al., 1996). 5′ and 3′ of the potential vimentin binding sequence are topoisomerase II sites, which are associated with regions of matrix attachment (Gasser et al., 1989, and references therein). Moreover, three T-box motifs (TTWTWTTWTT) are found between bp 5000 and 5590, which are also associated with matrix attachment (Gasser et al., 1989, and references therein). Nearby, there is a region with homology to Z-DNA motifs found in the nitric oxide synthase gene promoter, TCR V$\gamma$1 and V$\gamma$2 promoter, and the $\mu$-$\delta$ intron (Milstein et al., 1984; Kuziel et al., 1994; Eberhardt et al., 1996).

### Sequence Homologies with the RAG Intergenic Region of Other Species

Recently, the RAG intergenic region of the zebrafish and the trout have been sequenced (Bertrand et al., in press; Hansen, in press). A comparison of these sequences reveals that there is limited sequence homology between the teleost and the murine RAG intergenic regions (data not shown). This is perhaps not surprising because the intergenic region of the fish is also likely the 3′ UTR of RAG-1 and RAG-2 (Bertrand et al., 1997; Hansen, in press; Willett et al., 1997). However, no homologies were found between the murine RAG-1 and RAG-2 3′ UTR and the two teleost RAG intergenic regions. Moreover, the murine RAG-1 and RAG-2 3′ UTRs do not contain the wide array of Ig-related transcription-factor motifs observed in the intergenic region (data not shown).

Although there is limited homology among the mouse RAG 3′ UTRs, the mouse intergenic region, and the analogous region in the teleost, an area of the trout and zebrafish intergenic region share 50% similarity (Figure 3). This region contains motifs (mismatch = 1) for the transcription factors Cmu E4, CREB, Cmu E5, AP-1, E2A, Ets-1, and an enhancer core element, which are also found in the murine intergenic sequence. Thus, although there is no strong sequence homology between this region and murine RAG intergenic region, many of the potential regulatory sites are in common.

### DISCUSSION

The 7.0-kb murine RAG locus intergenic region has been sequenced and analyzed for potential *cis*-regulatory motifs. Several motifs known to be involved in the regulation of the IgH enhancer (reviewed in Ernst and Smale, 1995) and motifs involved in second messenger pathways that modulate RAG transcription (Menetski and Gellert, 1990; Casillas et al., 1995; Dobbeling et al., 1996) are contained within this sequence. However, preliminary

```
          ↓670      ↓680      ↓690      ↓700      ↓710      ↓720
Trout  TTTATTGTATTGCATGGGTTATGACACATCCATGAATTTATCCAGT-GGGTTTATTTATG
       TTT TTGTATTGC   GGTT TGACAC T CA GAATT AT CAG  GGGTT ATTTATG
Z-fish TTTTTTGTATTGCCA-GGTTGTGACACCTTCACGAATTCATGCAGAAGGGTT-ATTTATG
       ↑270      ↑280      ↑290      ↑300      ↑310      ↑320
           ↓730      ↓740      ↓750      ↓760      ↓770      ↓780
Trout  GGAACAAATGAAAGCACTGTAAGTCATGGAATGGAATTGTGATAAAGGAGC-TCACATTT
         AAC  A GAA G   TGTAAGTCA  GAA    ATT T  TAAAGGAGC TCAC TTT
Z-fish AAAACGTACGAAGGAGTTGTAAGTCACTGAA----ATTATATTAAAGGAGCCTCACGTTT
        ↑330      ↑340      ↑350          ↑360      ↑370      ↑380
            ↓790        ↓800      ↓810      ↓820      ↓830
Trout  ATGATCAGTGTATTAG--TTTATCTCCAGTCCTGGAGCAAAATGGAATCATATAGACTC-
       ATGAT   T TATTA   TTT  C  CAG     GAG  AAA  GAA     TAGA
Z-fish ATGATTGCTATATTAATATTTCACA-CAGGGACAGAGACAAAGTGAAAT---TAGAAAAT
            ↑390      ↑400       ↑410      ↑420      ↑430
                   ↓850      ↓860      ↓870             ↓880
Trout  ---AAATTGTCTTC--GTTTCAGGCTGCCATCTAAAAA-------TATTGCTTTAGAGCT
          AAATT  C     GTTT AGG           AAA       TATT  TTTA    T
Z-fish GTTAAATTTGCAGGAGGTTTGAGGG----------AAACAGGGGTTATT--TTTACCAAT
       ↑440      ↑450      ↑460                ↑470      ↑480
       ↓890      ↓900      ↓910      ↓920                ↓930
Trout  TCAT-ATTTGGGTCCTGAAGTCCAAAATGGGGGCCTG----GGT------GACTTTG---
        CAT ATTT      TGA         TGG GGC       GGT      GACT
Z-fish GCATTATTT------TGA---------TGGTGGCTCAAATKGGTCACAATGACTGAATGC
        ↑490                      ↑500      ↑510      ↑520      ↑530
            ↓940      ↓950      ↓960
Trout  AAACCATCTTTATCTGTTTTAGCATTTGCA
       AAA    C  TAT       AGCATTTGCA
Z-fish AAATAGACACTATGGTAC--AGCATTTGCA
             ↑540      ↑550         ↑560
```

FIGURE 3 The trout and zebrafish RAG intergenic regions contain a region of homology. Shown is a 290 base pair region of the zebrafish and trout RAG locus intergenic regions that is 50% similar. The middle line lists the base pairs in common. Numbering refers to accession numbers U73750 (trout) and U69610 (zebrafish).

reporter gene experiments with fragments of the intergenic region do not exhibit any enhancer activity in transient transfection assays (F. Bertrand and G. Wu, unpublished observations). Most enhancers and many promoters studied to date are associated with DNase I hypersensitive sites (Jenuwein et al., 1993, and references therein). Consistent with the lack of enhancing function in the RAG intergenic fragments, DNase hypersensitivity mapping of this locus has not identified any hypersensitive sites in the intergenic region (U. Storb, personal communication). However, this does not preclude this region from playing a modulatory role in the transcription of the RAG locus, in such a way that cannot be readily detected in transient reporter gene assays or revealed by DNase I hypersensitivity mapping.

Matrix attachment regions found within the immunoglobulin loci have been shown to play a role in the activation of the enhancers and transcriptional regulation of Ig gene segments (Cockerill and Garrard, 1986; Webb et al., 1991; Jenuwein et al., 1993). The region of the murine intergenic region from base pairs 4524 to 6908 may contain sites of matrix attachment, based on the presence of topoisomerase II sites and homology with the intermediate filament vimentin binding site. These proteins have been shown to be involved in the anchoring of DNA to regions of matrix attachment. Consistent with this idea is the presence of multiple copies of the motif (TTWTWTTWTT) in this region of the intergenic sequence. This "T-box" motif is also associated with matrix attachment regions (Gasser et al., 1989). The close proximity of a Z-DNA sequence motif, which may provide for an unusual DNA structure in this region, is also compelling. Thus, this region may contain regulatory features of the murine RAG locus that are based on DNA structure and accessibility. This type of regulation awaits a more detailed biochemical analysis.

Studies using fibroblasts transfected with genomic clones of the RAG locus and pre-B cell lines have demonstrated that overexpression of the transcription factor CREB results in a sharp increase of RAG transcription and the rearrangement of an exogenous plasmid substrate (Dobbeling et al., 1996). The CREB transcription factor is involved in cAMP responses (Gonzales and Montminy, 1989). In contrast, overexpression of both c-FOS and c-JUN in these cell lines decreased RAG transcription and rearrangement of an exogenous substrate. Because overexpression of c-FOS or c-JUN alone had no effect, the repression of RAG transcription mediated by these two transcription factors likely involves AP-1 sites, which bind the FOS/JUN heterodimer (Dobbeling et al., 1996). For these reasons, we were intrigued to find AP-1 sites and a single CREB site in the RAG-locus intergenic sequence. It is possible that these sites participate in the cAMP and PKC second messenger pathways that have been shown to modulate RAG-1 and RAG-2 transcription.

Sequence comparisons of the murine RAG 3′ UTRs and the intergenic region with the RAG intergenic regions of the zebrafish and trout indicate that these sequences in between the RAG genes have not been conserved during evolution, although the RAG coding regions have been. However, this circumstance does not mean that function is not preserved between these species. For instance, there is little sequence homology between the trout and mouse IgH enhancers, yet the respective regions of DNA in the two species have the same function (Magor et al., 1994). In both the zebrafish (Bertrand et al., in press) and in the mouse, there are potential binding sites for transcription factors that act in response to second messenger pathways, such as cAMP. An ~200-bp region of homology between the trout and zebrafish intergenic regions is compelling in that perhaps this sequence was preserved due to some common function. This region also contains many of the same transcription-factor motifs found in the murine intergenic sequence, suggesting a common regulatory function in the mouse and teleost.

### *Acknowledgements*

The sequence reported here has been deposited in GenBank under the accession number U96151.

### *References*

Almendro N., Bellon T., Rius C., Lastres P., Langa C., Corbi A. and Bernabeu C. (1996) Cloning of the human platelet endothelial cell adhesion molecule-1 promoter and its tissue specific expression. *J. Immunol.*, **157**: 5411-5421.

Bertrand III F.E., Olson S.L., Willett C.E. and Wu G.E. (In press) Sequence of the rag1 and rag2 intergenic region in zebra fish (*Danio rerio*). *Dev. Immunol.*

Casillas A.M., Thompson A.D., Cheshier S., Hernandez S. and Aguilera R.J. (1995) RAG-1 and RAG-2 gene expression and V(D)J recombinase activity are enhanced by protein phosphotase 1 and 2A inhibition in lymphocyte cell lines. *Mol. Immunol.*, **32**: 167-175.

Chun J.J.M., Schatz D.G., Oettinger M.A., Jaenisch R. and Baltimore D. (1991) The recombinase activating gene-1 (RAG-1) transcript is present in the murine central nervous system. *Cell*, **64**: 189-200.

Cockerill P.N. and Garrard W.T. (1986) Chromosomal loop anchorage of the kappa immunoglobulin gene occurs next to the enhancer in a region containing topoisomerase II sites. *Cell*, **44**: 273-282.

Dobbeling U., Hobi R., Berchtold M.W. and Kuenzle C.C. (1996) V(D)J recombination is regulated similarly in RAG-transfected fibroblasts and pre-B cells. *J. Mol. Biol.*, **261**: 309-314.

Eberhardt W., Kunz D., Hummel R. and Pfeilschifter J. (1996) Molecular cloning of the Rat inducible nitric oxide synthase gene promoter. *Biochem. Biophys. Res. Comm.*, **223**: 752-756.

Ernst P. and Smale S.T. (1995) Combinatorial regulation of transcription II: The immunoglobulin mu heavy chain gene. *Immunity*, **2**: 427-438.

Gasser S.M., Amati B.B., Cardenas M.E. and Hofmann J.F.-X. (1989) Studies on scaffold attachment sites and their relation to genome function. *Int. Rev. Cytol.*, **119**: 57-96.

Gebhard W., Meitinger T., Hochtl J. and Zachau H.G. (1972) A new family of interspersed repetitive DNA sequences in the mouse genome. *J. Mol. Biol.*, **157**: 453-471.

Gebhard W. and Zachau H.G. (1983) Organization of the R family and other interspersed repetitive DAN sequences in the mouse genome. *J. Mol. Biol.*, **170**: 255-270.

Ghia P., ten Boekel E., Sanz E., de la Hera A., Rolink A. and Melchers F. (1996) Ordering of human bone marrow B lymphocyte precursors by single-cell polymerase chain reaction analysis of the rearrangement status of the immunoglobulin H and L chain gene loci. *J. Exp. Med.*, **184**: 2217-2229.

Gonzales G.A. and Montminy M.R. (1989) Cyclic cAMP stimulates somatostatin gene transcription by phosphorylation of CREB at serine 133. *Cell*, **59**: 675-680.

Han S., Zheng B., Schatz D.G., Spanopoulo E. and Kelsoe G. (1996) Neoteny in lymphocytes: RAG1 and RAG2 expression in germinal center B cells. *Science*, **274**: 2094-2097.

Hansen J. D. (In press). Inspection of the 3′ UTR genomic region for RAG1 and 2 in rainbow trout (*Oncorhynchus mykiss*) reveals potential regulatory motifs. *Dev. Immunol.*

Hikida M., Mori M., Takai T., Tomochika K-I., Hamatani K. and Ohmori H. (1996) Reexpression of RAG-1 and RAG-2 gene in activated mature mouse B cells. *Science*, **274**: 2092-2094.

Jenuwein T., Forrester W.C., Qiu R.-G. and Grosschedl R. (1993) The immunoglobulin mu enhancer core establishes local factor access in nuclear chromatin independent of transcriptional stimulation. *Genes and Dev.*, **7**: 2016-2032.

Kuziel W.A., Kienker L.J. and Tucker P.W. (1994) Physical linkage of mouse Tcrg-V genes I. Evolutionary and regulatory features of the Vg1.1-Vg1.2 intergenic region. *Immunogenetics*, **39**: 296-297.

Lewis S.M. (1994) The mechanism of V(D)J joining: Lessons from molecular, immunological and comparative analysis. *Adv. Immunol.*, **56**: 27-150.

Li Y., Hayakawa K. and Hardy R.R. (1993) The regulated expression of B lineage associated genes during B cell differentiation in bone marrow and fetal liver. *J. Exp. Med.*, **178**: 951-960.

Magor B.G., Wilson M.R., Miller N.W., Clem L.W., Middleton D.L. and Warr G.W. (1994) An Ig heavy chain enhancer of the channel catfish *Ictalurus punctatus*: Evolutionary conservation of function not structure. *J. Immunol.*, **53**: 5556-5563.

Menetski J.P. and Gellert M. (1990) V(D)J recombination activity in lymphoïd cell lines is increased by agents that elevate cAMP. *Proc. Natl. Acad. Sci. USA*, **87**: 9324-9328.

Milstein C.P., Deverson E.V. and Rabbitts T.H. (1984) The sequence of the human immunoglobulin $\mu$-$\delta$ intron reveals possible vestigial switch segments. *Nuc. Acids Res.*, **16**: 6523-6535.

Mombaerts P., Iacomini J., Johnson R.S., Herrup K., Tonegawa S. and Papaioannou V.E. (1992) RAG-1 deficient mice have no mature B and T lymphocytes. *Cell*, **68**: 869-877.

Oettinger M.A., Shatz D.G., Gorka C. and Baltimore D. (1990) RAG-1 and RAG-2, adjacent genes that synergistically activate V(D)J recombination. *Science*, **248**: 1517-1523.

Schatz D.G., Oettinger M.A. and Baltimore D. (1989) The V(D)J recombination activating gene, RAG-1. *Cell*, **59**: 1035-1048.

Selvakumar A., Mohanraj B.K., Eddy R.L., Shows T.B., White P.C. and Dupont B. (1992) Genomic organization and chromosomal location of the human gene encoding the B-lymphocyte activation antigen B7. *Immunogenetics*, **36**: 175-181.

Shinkai Y., Rathbun G., Lam K.-P., Oltz E.M., Stewart V., Mendelsohn M., Charron J., Datta M., Young F., Stall A.M. and Alt F.W. (1992) RAG-2 deficient mice lack mature lymphocytes owing to inability to initiate V(D)J rearrangement. *Cell*, **68**: 855-867.

Takeda S., Masteller E.L., Thompson C.B. and Buerstedde J.M. (1992) RAG-2 expression is not essential for chicken immunoglobulin gene conversion. *Proc. Natl. Acad. Sci. USA*, **89**: 4023-4027.

Wang X., Tostonog G., Shoeman R.L. and Traub P. (1996) Selective binding of specific mouse genomic DNA fragments by mouse vimentin filaments in vitro. *DNA Cell Biol.*, **15**: 209-225.

Webb C.F., Das C., Eneff K.L. and Tucker P. W. (1991) Identification of a matrix-associated region 5′ of an immunoglobulin heavy chain variable region gene. *Mol. Cell. Biol.*, **11**: 5206-5211.

Willett C.E., Chery J.J. and Steiner L.A. (1997) Characterization and expression of the recombination activating genes (rag1 and rag2) of Zebrafish. *Immunogenetics*, **45**, 394–404.

